# The Humanistic and Economic Burden of Restless Legs Syndrome

**DOI:** 10.1371/journal.pone.0140632

**Published:** 2015-10-26

**Authors:** Tracy Durgin, Edward A. Witt, Jesse Fishman

**Affiliations:** 1 UCB Pharma, Smyrna, Georgia, United States of America; 2 Kantar Health, Princeton, New Jersey, United States of America; University of Rome Tor Vergata, ITALY

## Abstract

**Objectives:**

To evaluate the humanistic and economic burden of a restless legs syndrome (RLS) diagnosis with regard to health-related quality of life, work productivity loss, healthcare resource use, and direct and indirect costs.

**Study Design:**

Self-reported data came from the 2012 National Health and Wellness Survey (NHWS), a large, annual, nationally representative cross-sectional general health survey of US adults.

**Methods:**

RLS patients (n = 2,392) were matched on demographic and health characteristics to Non-RLS respondents via propensity score matching differences between groups were tested with Bivariate and multivariable analyses.

**Results:**

RLS patients had significantly lower health-related quality of life scores: Mental Component Summary (44.60 vs. 48.92, *p*<.001), Physical Component Summary (40.57 vs. 46.78, *p*<.001), Health Utilities (.63 vs. .71, *p*<.001) and higher levels of work productivity loss in the past seven days including absenteeism (8.1% vs. 9.3%, *p*<.001), presenteeism (26.5% vs. 15.8%, *p*<.001), and overall productivity loss (30.1% vs. 18.1%, *p*<.001) as well as general activity impairment (46.1% vs. 29.7%, *p*<.001). RLS patients had significantly higher healthcare resource use in the past 6 months than non-RLS patients: healthcare provider visits (7.46 vs. 4.42%, *p*<.001), ER visits (0.45 vs. 0.24, *p*<.001), and hospitalizations (0.24 vs. 0.15, *p*<.001). RLS patients also had higher estimated direct and indirect costs than non-RLS patients. Finally, it was found that across outcomes increasing severity is associated with increased economic and humanistic burden for RLS patients.

**Conclusions:**

RLS patients suffer a greater humanistic and economic burden than those without RLS. Moreover as severity increases so does the burden of RLS.

## Introduction

Restless legs syndrome (RLS)/WED is a sensorimotor disorder defined by a strong urge to move the legs usually associated with unpleasant sensations in the legs. [1] A review of RLS epidemiology studies from North America and Western Europe—comprising over 230,000 participants— reported a prevalence ranging from 4% to 29%.[2] Primary RLS (i.e., RLS not secondary to another pathology, such as iron deficiency or end-stage renal disease) is a common neurological disorder. Prevalence estimates range from 1.5–10% in industrialized countries, though are slightly higher in certain rural populations (e.g., U.S. Appalachia at 10.1–19.6%) and increases with age.[3–7] Representative US panel data estimate 1.5% of primary RLS sufferers experience symptoms at least twice per week (termed moderate to severe RLS), with women most likely to experience severe symptoms.[5,7] Despite its notable prevalence, less than 41% of severely-symptomatic patients report an actual physician diagnosis of RLS.[5]

Consequences of RLS include discomfort, pain, inability to rest, sleep loss, difficulties sitting/relaxing, decreased physical functioning, psychological distress, and lowered cognitive function—all of which are associated with reduced health-related quality of life (HR-QoL).[8,9] Patients with RLS show physical and mental health HR-QoL scores more than a standard deviation lower than sex- and age-adjusted general population norms.[10,11] Moreover, RLS patients show poorer HR-QoL than those with other chronic medical and psychiatric conditions, such as hypertension, type II diabetes, osteoarthritis, and depression.[2,4] Patients with untreated RLS show significantly higher depressive symptoms than treated patients and healthy controls.[12] Strikingly, male RLS patients show elevated mortality rates, independent of known risk factors.[13] RLS-related burden has been shown to increase with symptom severity, symptom frequency, and level of symptom-related distress.[5,10,14,15] For example, frequent symptoms (i.e., occurring at least two days per week) have been associated with impaired frontal lobe executive function (e.g., thinking about abstract concepts, attention, and verbal function).[16,17] These types of impairments are thought to relate to an increase in RLS-related sleep disturbances with greater severity.

A systematic literature review found that compared with matched controls, RLS patients show significantly higher health expenditures from a third-party payer’s perspective.[4] Indirect costs due to productivity loss, with loss as high as 20% of normal productive capacity, are also thought to be a large contributor to the economic burden associated with RLS.[2] Patients with symptoms at least two days per week and moderate-to-severe distress have shown mean productivity loss of one entire day per week, reported as directly-attributable to RLS.[5] This degree of productivity loss for RLS patients is similar to reports of productivity loss for diabetes with neuropathy, or bipolar disorder, and is thought to relate to RLS-associated sleep disturbances.[18–20]

RLS-related burden appears to be significant, but further research is needed to substantiate and clearly define the economic burden of RLS.[8] Existing economic studies, for example, have relied on claims data or did not account for potential confounding variables. Furthermore, U.S. studies have not included comparisons to control cohorts. In addition, there has been little U.S. research reported investigating the economic burden of RLS as a function of disease severity. Given this need for further research, the objectives of the current study were to examine the humanistic and economic effects of an RLS diagnosis and to evaluate whether or not increased RLS severity is associated with worse outcomes. To answer this question, we contrasted individuals with RLS and a matched comparison of patients not reporting a diagnosis of RLS across a variety of health outcomes including health-related quality of life, work productivity, healthcare resource use, and direct and indirect costs. For those with an RLS diagnosis, we also compared these outcomes across symptom severity groups (mild, moderate, and severe).

## Methods

### Data Source and Analysis Sample

Data were drawn from the 2012 US National Health and Wellness Survey (NHWS; n = 71,141) a self-administered, annual, internet-based questionnaire of US adults. The NHWS is fielded through an opt-in panel using a random stratified sampling framework to match the demographic composition of the United States adult population (18+). Previous suggests that NHWS data compares favorably with estimates of U.S. demographic composition according to the census as well as estimates from the National Health Interview Survey.[21–23] The survey was approved by the Essex Institutional Review Board (Lebanon, NJ).

### Measures

#### RLS Diagnosis and Severity

Restless Leg Syndrome (RLS) Diagnosis: Patients were presented with a list of medical conditions and asked to indicate which conditions they had ever experienced. If they selected “restless legs syndrome” from the list, they were asked “Has your Restless Legs Syndrome been diagnosed by a physician?” If the patient answered “yes”, he or she was categorized as “RLS” (n = 2,392) all other patients were categorized as “Non-RLS”.

RLS Severity: RLS patients also self-reported their current perceived RLS severity as either “mild” (n = 904), “moderate” (n = 1,130), or “severe” (n = 358) via one question.

#### Outcome Measures

Health-Related Quality of Life (HR-QoL): HRQoL was measured using the short form health survey version 2 (SF-36v2), specifically, the mental and physical component summary scores (MCS and PCS, respectively) and health utility score. MCS and PCS scores are normalized based on the US population with mean of 50 indicating the health of an average US citizen and a standard deviation of 10. Higher scores indicate better health status. The health utility score has interval scoring properties and yields summary scores on a theoretical 0–1 scale. Higher scores indicate better health status.[24]

Labor Force Participation and Work Productivity Loss: Labor force participation was measured via one item: “*What is your employment status*?” Responses of “employed full time”, “employed part-time”, or “self-employed” were categorized as “employed”. Work productivity loss and activity impairment were measured via the Work Productivity and Activity Impairment scale (WPAI-GH).[25]

Disability Status: Patients were categorized into one of three disability statuses: long-term, short-term, and not disabled based on the responses from the employment status question.

Healthcare Resource Use: Healthcare resource use was measured with three questions asking patients to report the number of times they used three different forms of healthcare resources (ER visits, hospitalizations, and physician visits) over the past 6 months.

Costs: Estimated healthcare resource use costs were calculated by extrapolating data from the Medical Expenditure Panel Survey (MEPS)[26] to apply as unit costs to healthcare resource use variables from the NHWS. Estimated wages were obtained from the Bureau of Labor Statistics (BLS)[27] and applied to work productivity impairment measures to estimate indirect costs.

Demographics measured included age, gender, race/ethnicity, marital status, education (university degree vs. <university degree), household income, body mass index (BMI), smoking status (current, former, never), alcohol use (current vs. not current), exercise behavior (currently exercise vs. not currently exercise), Charlson comorbidity index (CCI).[28]

### Statistical analysis

RLS/Non-RLS: Pre-match differences between groups (RLS/Non-RLS) were assessed via Chi-square tests and one-way ANOVAs. RLS patients were then matched to Non-RLS patients via a 1:1 propensity score match: First, a logistic regression model where RLS/Non-RLS was regressed on all measured demographic and health characteristics was conducted and the predicted scores (i.e., propensity scores) for each individual were calculated. Next, target individuals were matched via a greedy algorithm to Non-RLS patients based on propensity scores. Finally, differences between the newly matched groups on covariates used in the match were tested inferentially (using Chi-Square tests and one-way ANOVAs), variables that remained significant post-match were controlled for in subsequent analyses.

RLS Severity: Bivariate associations between RLS Severity (Mild, Moderate, Severe) and outcomes were calculated using Chi-square tests and one-way ANOVAs. For multivariable comparisons, general linear- and generalized linear models were used to assess differences in health outcomes across levels of severity holding covariates constant. The type of model and link function was dictated by the distribution of the specific variable under examination.

## Results

### Pre-Match Analyses

RLS patients (n = 2,392) were older (55.99 vs. 55.07, *p*<.05), and high proportions reported being female (56.9% vs. 50.5%, *p*< .001), non-Hispanic white (83.9% vs. 71.1%, *p*< .001), married (60.7% vs. 58.2%, *p* = .012), without a college degree (44.4% vs. 51.7%, *p*<.001), lower incomes (% < $50k = 54.6% vs. 43.2%, *p*<.001), higher BMIs (% Obese = 47.1% vs. 31.4%, *p*<.001), and being a current or former smoker (62.7% vs. 46.2%, *p*<.001) compared to non-RLS comparison patients. Fewer of them were also currently pregnant (.4% vs. .9%, *p* = .007), currently drinking alcohol (56.7% vs. 64.3%, *p*<.001), and had exercised in the past thirty days (52.3% vs. 65.6%, *p*<.001). In addition, RLS patients had a significantly higher comorbidity burden than non-patients (Mean CCI = 1.01 vs. .36, *p*<.001). All of these variables were included in the propensity score matching model.

### Post-Match Analyses

Two RLS patients could not be matched to a compatible propensity score in the pool of non-RLS participants. This left two equally sized groups for comparison (n = 2,390 each). Race/ethnicity, BMI, and exercise status were still statistically significantly different between the groups post-match and were controlled for in subsequent analyses.

Compared to matched non-RLS patients, RLS patients had significantly lower MCS (44.60 vs. 48.92, *p*<.001), PCS (40.57 vs. 46.78, *p*<.001) and Health Utilities scores (.63 vs. .71, *p*<.001). These differences are considerable given that a difference of 3 points on the MCS and 2 points on the PCS scores constitute a “minimally important difference”.[24]

**Fig 1:**
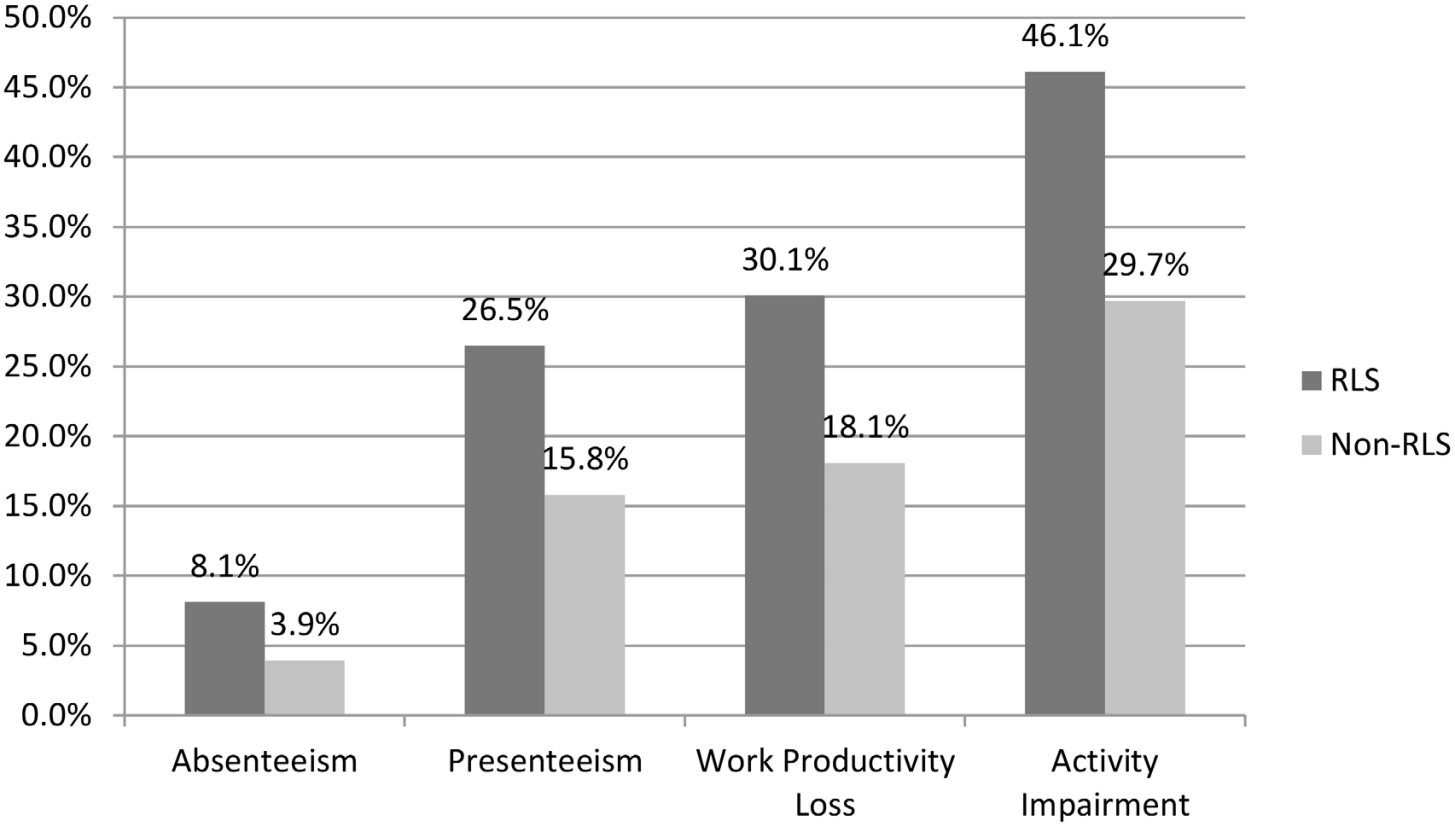
Percentage Work Productivity and Activity Impairment (past 7 days) by RLS Status. *Note*:Values represent least square means controlling for significant post-match differences on Race/Ethnicity, BMI, and Exercise Status. All comparisons significant at *p* < .001.

Fewer RLS patients were employed (34% vs. 43%, *p*<.001) and more were on short or long-term disability (15% vs. 7%, *p*<.001) than matched non-RLS patients. Across all work productivity metrics examined, RLS patients experienced significantly higher work productivity loss than non-RLS comparison patients (see Fig 1). RLS patients also reported significantly more activity impairment in their day-to-day lives than non-RLS patients (Fig 1).

**Fig 2:**
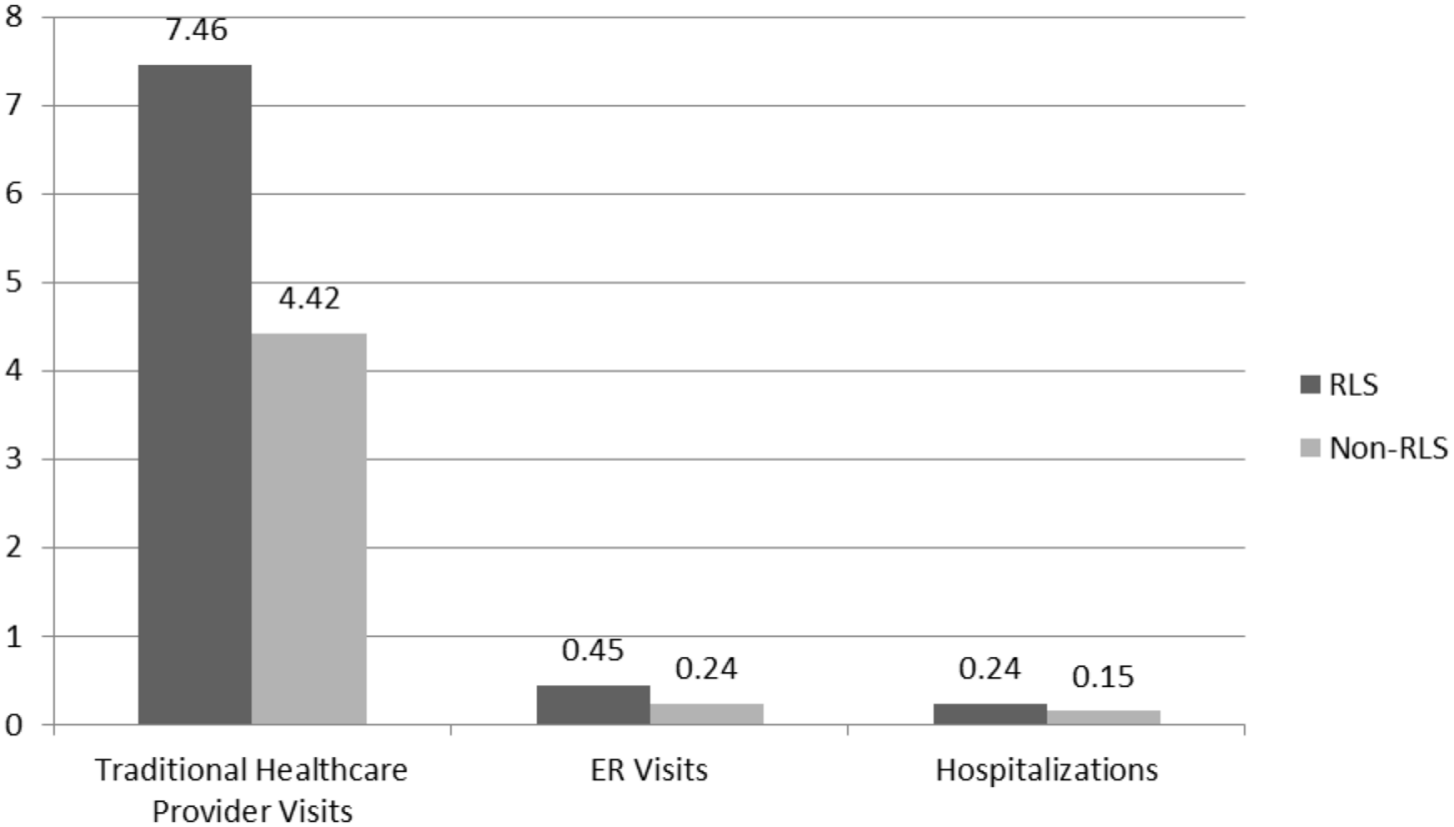
Healthcare Resource use by RLS Status (# of visits past 6 months). *Note*:Values represent least square means controlling for significant post-match differences on Race/Ethnicity, BMI, and Exercise Status. All comparisons significant at *p* < .001.

**Fig 3:**
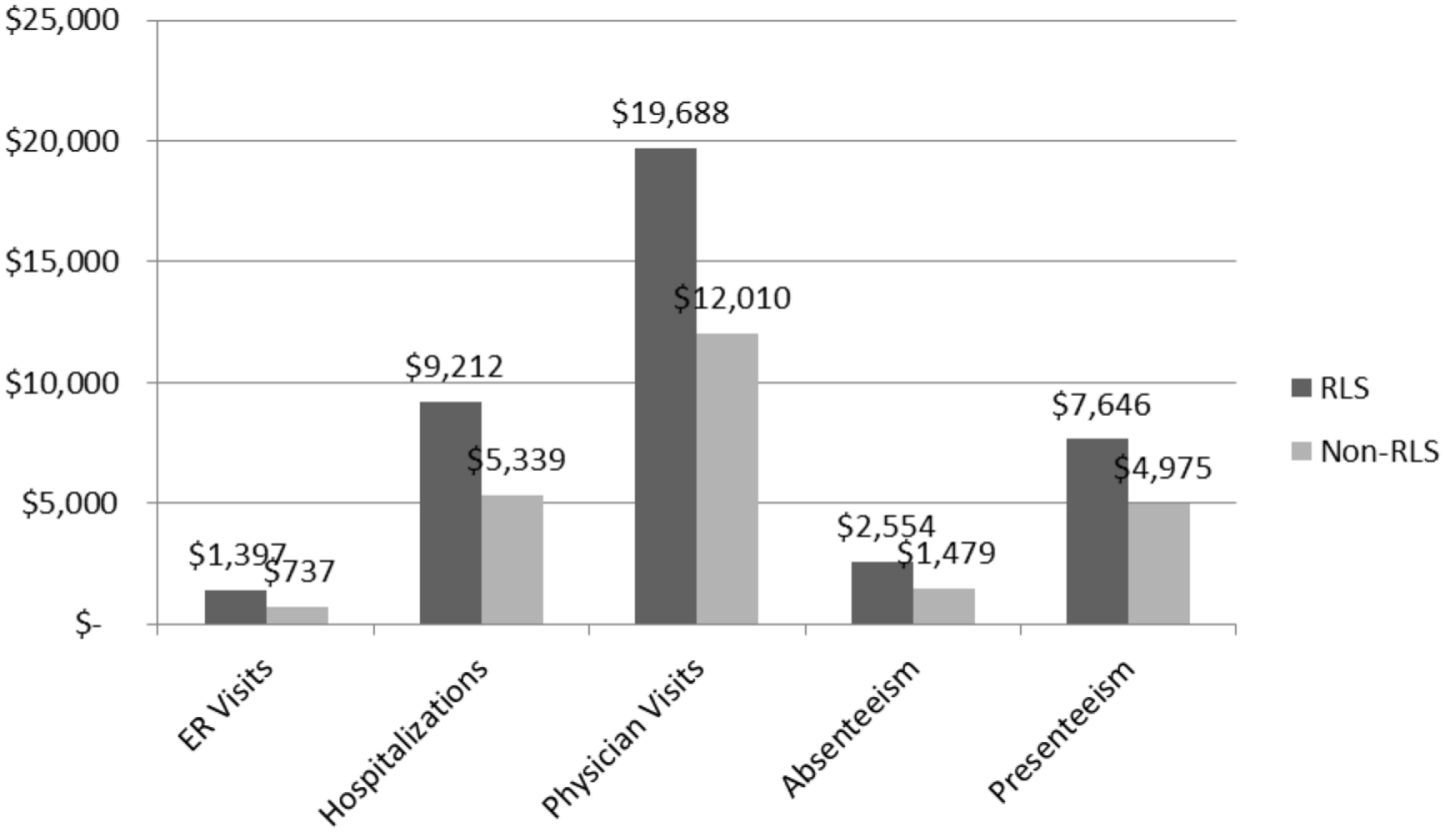
Estimated Annualized Direct and Indirect Costs by RLS Status. *Note*:Values represent least square means controlling for significant post-match differences on Race/Ethnicity, BMI, and Exercise Status. All comparisons significant at *p* < .001.

As can be seen in Fig 2, RLS also reported statistically significantly more healthcare resource use across use categories than matched non-RLS patients. Relatedly, RLS responds had significantly higher estimated annual costs for ER visits, hospitalizations, and physician visits, resulting in significantly higher overall direct healthcare resource costs (Fig 3). Similarly, RLS patients had significantly higher indirect costs from absenteeism and presenteeism than matched non-RLS patients (Fig 3).

### Severity Analyses

The analyses above using the same outcomes (quality of life, work productivity, and healthcare resource use, direct and indirect costs) were replicated within RLS patients to determine the effect of severity (mild, moderate, severe) on these outcomes controlling for covariates.

There were several demographic differences that were significantly associated with RLS severity. Specifically, as severity increased, so did age (Mean age: Mild = 55.20, Moderate = 56.59, Severe = 56.01), the proportion of female patients (Mild = 54.2%, Moderate = 56.8%, Severe = 63.7%), and comorbidity burden (Mean CCI: Mild = .90, Moderate = 1.07, Severe = 1.73). In contrast, as severity increased, income decreased (% ≤ $50k: Mild = 49.2%, Moderate = 56.7%, Severe = 61.5%), as did the proportion currently consuming alcohol (Mild = 61.5%, Moderate = 54.5%, Severe = 51.4%), the proportion with a college degree (Mild = 48.6%, Moderate = 43.8%, Severe = 35.5%), and the proportion of patients who have exercised in the past 30 days (Mild = 58.6%, Moderate = 49.3%, Severe = 45.8%). Race/ethnicity, marital status, BMI, smoking status, and pregnancy status did not differ significantly by severity. To adjust for confounding and better isolate the unique association of severity with health outcomes, all of the above variables, regardless of whether or not they differed statistically between groups, were controlled for in subsequent analyses. Table 1 contains estimated least square means controlling for covariates for all health outcomes examined.

**Table 1:** Health Outcomes by RLS Severity.

	Non-RLS		Mild		Moderate		Severe		
	M	95% CI	M	95% CI	M	95% CI	M	95% CI	*p*_trend_
Health-Related Quality of Life									
Mental Component Summary	49.17	(49.10–49.24)	46.73	(46.08–47.37)	44.08	(43.50–44.66)	42.04	(41.02–43.07)	< .001
Physical Component Summary	50.52	(50.46–50.58)	45.63	(45.08–46.17)	43.46	(42.97–43.95)	41.71	(40.84–42.58)	< .001
Health Utilities	.737	(.736-.738)	.676	(.667-.684)	.644	(.637-.652)	.615	(.602-.628)	< .001
Labor Force Participation	.54	(.53-.54)	.48	(.45-.51)	.44	(.41-.46)	.46	(.41-.51)	.001
Disabled	.04	(.04-.04)	.08	(.07-.09)	.12	(.11-.14)	.18	(.16-.20)	< .001
Work Productivity/Activity Impairment									
Absenteeism	3.48	(3.33–3.63)	5.81	(4.29–7.33)	8.62	(7.07–10.16)	10.05	(7.33–12.77)	< .001
Presenteeism	12.83	(12.60–13.07)	21.86	(19.48–24.23)	26.33	(23.91–28.75)	30.00	(25.71–34.29)	< .001
Overall Work Productivity Loss	14.90	(14.64–15.17)	24.55	(21.88–27.23)	30.19	(27.47–32.91)	34.17	(29.38–38.96)	< .001
Activity Impairment	21.57	(21.37–21.78)	33.33	(31.52–35.13)	41.09	(39.48–42.70)	45.98	(43.16–48.79)	< .001
Healthcare Resource Use									
ER Visits	.18	(.18-.19)	.37	(.31-.43)	.39	(.34-.45)	.50	(.40-.59)	< .001
Hospitalizations	.11	(.10-.11)	.20	(.14-.26)	.19	(.14-.24)	.25	(.15-.34)	< .001
HCP Visits	3.43	(3.39–3.47)	5.92	(5.57–6.28)	6.31	(5.99–6.63)	7.57	(7.01–8.13)	.006

*Note*: Values represent least square means controlling for significant differences on age, gender, income, alcohol status, college degree status, and exercise status. ER = Emergency Room, HCP = Healthcare provider, M = Mean, 95% CI = 95% confidence interval.

As RLS symptom severity increased from mild to severe RLS, estimated means for health related quality of life decreased (Table 1). These trends were significant (all *p*<.001) for MCS, PCS, and Health Utility scores. Moreover, all levels of RLS severity were associated with lower health related quality of life scores relative to individuals with no symptoms at all (non-RLS). Similar to health related quality of life, increases in severity were associated with increases in likelihood of short or long term disability and decreased likelihood of labor force participation. Those without RLS (non-RLS) had significantly higher levels of employment and lower levels of disability than all levels of RLS severity.

Severity level was also significantly associated with work productivity loss and activity impairment such that individuals with higher levels of severity reported higher estimated levels of work productivity loss and impairment and in all cases productivity loss and impairment were higher for those with RLS symptoms than those without (Table 1).

**Fig 4:**
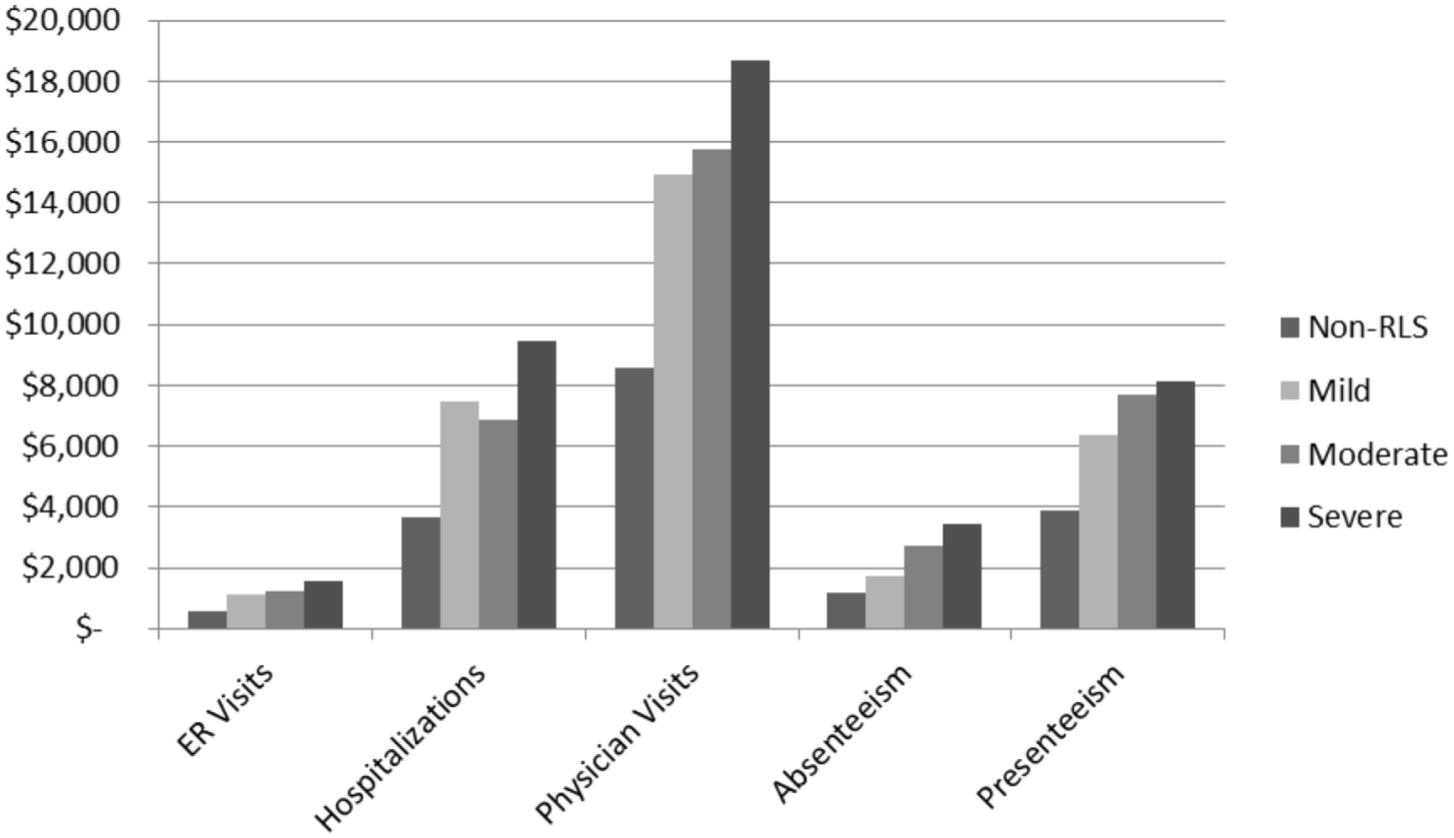
Estimated Direct and Indirect Costs by RLS Severity. Values represent least square means controlling for significant differences on age, gender, income, alcohol status, college degree status, and exercise status. ER = Emergency Room, HCP = Healthcare provider, M = Mean, 95% CI = 95% confidence interval.

Finally, severity was also associated with subsequently higher estimates of traditional health care provider visits, ER visits, and hospitalizations (Table 1). Relatedly, estimates of direct and indirect costs also increased as severity increased (Fig 4).

## Discussion

This study illustrates the magnitude of the humanistic and economic burden associated with RLS and lends further insight into the effect of RLS severity on these outcomes. To our knowledge, this is the most comprehensive survey study to describe RLS-associated humanistic and economic burden across these domains. Consistent with the available literature, patients reporting an RLS diagnosis experienced significantly worse health outcomes, including lower HR-QoL, and more healthcare resource use than comparison individuals. Diagnosed patients reported significantly more work productivity loss (both for absenteeism and presenteeism) and overall activity impairment than their matched counterparts. Furthermore, it appears that RLS diagnosis places a significant financial burden on those diagnosed and the healthcare system, as diagnosed patients had higher estimated direct, indirect, and total costs of healthcare resource use compared to comparison individuals. This finding corroborates other available estimates in the literature. For example, in a large U.S. managed care database study, 91% of patients had one or more RLS-related office visits, and patients filled an average of 6.5 RLS-related prescriptions during a one-year follow-up period, with opioids being the most common adjunctive treatment. RLS-related health care costs were 6.7% of total all-cause health care costs [M = $774(SD = $1,504)]. Furthermore, higher symptom frequency/degree of disease-related distress may relate to increasing economic costs.[5,8] For example, the mean direct annualized costs of RLS-specific healthcare resources/medications were estimated at USD $350.54 per patient—with an increase to $490.70 for patients with symptoms at least two days per week and moderate-to-severe distress.[5] This effect is striking when considered in the context of estimated costs for other diseases (e.g., bipolar disorder and asthma)[29] It should be noted, however, that the overall estimates found here are higher than those previously reported which was likely due to the fact that we annualized general healthcare resource use as opposed to the RLS-specific healthcare resource use. Finally, in line with the small body of previous literature, the degree of RLS symptom severity was found to be associated with an increase in the humanistic and economic burden of RLS.[2,5,10,14,15]

Strengths of this study included the recency of the data, the sample size relative to previous work, and the use of a propensity score matching approach. Such an approach allowed for the comparison of groups based on measured confounders, which is essential in a cross-sectional study. Data were self-reported so the limitations self-report (e.g., recall bias) applied to this study and it was not possible to verify responses (e.g., diagnosis) via some other means (e.g., physician assessment). Moreover, as the NHWS is a broad general health survey, it did not include diagnostic criteria or validated instruments for measuring RLS diagnosis and severity. The degree to which this may have affected the results is unclear and these results should be interpreted with these caveats in mind. The NHWS is a cross-sectional study and this analysis was restricted to U.S. respondents—therefore, only correlational inferences to be made and the results may not generalize to ex-U.S. populations. We chose an a priori significance cutoff of *p* < .05 for all our conducted analyses, given this and the number of comparisons conducted there is a higher likelihood of Type 1 error in these results than if we had chosen a smaller *p* critical value and/or conducted fewer comparisons. Previous research studies have eliminated conditions that might be mistaken for RLS, such as end-stage renal disease from analyses. As the NHWS is a general survey, we were restricted to the conditions surveyed and were thus limited in our ability to eliminate these conditions. It is reasonable to speculate that, were we able to remove those patients from the analyses the estimated burden of RLS may have been lower than what was found in these analyses. Finally, this study adjusted for measured confounders but results could have been affected by non-measured confounders.

## Conclusion

An RLS diagnosis confers a heavy humanistic and economic burden. This burden increases with symptom severity and is highest in certain demographic groups. The burden affects diagnosed individuals across a range of health domains. This strain may be directly and indirectly affecting the work force, the healthcare system, and individuals’ finances—and may be worse in those with severe symptoms—who may also have lower income levels to begin with. Improved management of RLS symptoms may offset this degree of distress, dysfunction, and disability endured by patients and effects on economic and health care systems.
