# Correction: The Humanistic and Economic Burden of Restless Legs Syndrome

**DOI:** 10.1371/journal.pone.0145139

**Published:** 2015-12-10

**Authors:** Tracy Durgin, Edward A. Witt, Jesse Fishman

There is an error in the first sentence of the Results section in the Abstract. The correct sentence is: RLS patients had significantly lower health-related quality of life scores: Mental Component Summary (44.60 vs. 48.92, p<.001), Physical Component Summary (40.57 vs. 46.78, p<.001), Health Utilities (.63 vs. .71, p<.001) and higher levels of work productivity loss in the past seven days including absenteeism (8.1% vs. 3.9%, p<.001), presenteeism (26.5% vs. 15.8%, p<.001), and overall productivity loss (30.1% vs. 18.1%, p<.001) as well as general activity impairment (46.1% vs. 29.7%, p<.001).

There are errors in the first sentence of the Pre-Match Analyses section of the Results. The correct sentence is: RLS patients (n = 2,392) were older (55.99 vs. 55.07, p<.05), and high proportions reported being female (56.9% vs. 50.5%, p<.001), non-Hispanic white (83.9% vs. 71.1%, p<.001), married (60.7% vs. 58.2%, p = .012), without a college degree (55.6% vs. 48.3, p<.001), lower incomes (% < $50k = 54.6% vs. 43.2%, p<.001), higher BMIs (% Obese = 47.1% vs. 31.4%, p<.001), and being a current or former smoker (62.7% vs. 46.2%, p<.001) compared to non-RLS comparison patients.
